# Anticipatory Adjustments to Being Picked Up in Infancy

**DOI:** 10.1371/journal.pone.0065289

**Published:** 2013-06-20

**Authors:** Vasudevi Reddy, Gabriela Markova, Sebastian Wallot

**Affiliations:** 1 University of Portsmouth, Portsmouth, United Kingdom; 2 Academy of Sciences of the Czech Republic, Prague, Czech Republic; 3 Interacting Minds Center, Aarhus University, Aarhus, Denmark; Royal Holloway, University of London, United Kingdom

## Abstract

Anticipation of the actions of others is often used as a measure of action understanding in infancy. In contrast to studies of action understanding which set infants up as observers of actions directed elsewhere, in the present study we explored anticipatory postural adjustments made by infants to one of the most common adult actions directed to *them* – picking them up. We observed infant behavioural changes and recorded their postural shifts on a pressure mat in three phases: (i) a prior Chat phase, (ii) from the onset of Approach of the mother’s arms, and (iii) from the onset of Contact. In Study 1, eighteen 3-month-old infants showed systematic global postural changes during Approach and Contact, but not during Chat. There was an increase in specific adjustments of the arms (widening or raising) and legs (stiffening and extending or tucking up) during Approach and a decrease in thrashing/general movements during Contact. Shifts in postural stability were evident immediately after onset of Approach and more slowly after Contact, with no regular shifts during Chat. In Study 2 we followed ten infants at 2, 3 and 4 months of age. Anticipatory behavioural adjustments during Approach were present at all ages, but with greater differentiation from a prior Chat phase only at 3 and 4 months. Global postural shifts were also more phase differentiated in older infants. Moreover, there was significantly greater gaze to the mother’s hands during Approach at 4 months. Early anticipatory adjustments to being picked up suggest that infants’ awareness of actions directed to the self may occur earlier than of those directed elsewhere, and thus enable infants’ active participation in joint actions from early in life.

## Introduction

The present study explores infants’ anticipation of one of the most common experiences of actions which adults direct towards them – picking them up. Anticipation is seen as a more stringent measure of infant awareness than habituation/looking time or preferential looking methods, since it requires real-time prediction in live ongoing interaction [1], [2]. While habituation/looking time methods have revealed infant discrimination of the goal-directedness of the actions of human agents at 5 months [3], [4] and even at 3 months [5], studies using anticipatory gaze have shown action prediction and anticipation of others’ intentional actions only later at 6, 10 and 12 months [6], [7], [8]. In all of these studies infants are asked to observe simple human actions directed to target objects. Infant experience from birth, however, is predominantly of human actions directed towards *themselves*. It is plausible then that infant anticipation of actions directed towards themselves would be evident earlier than of actions directed towards other target objects. Such a prediction is strengthened by evidence that others’ attention directed to self is preferred and arouses appropriate responses much earlier than attention directed towards other targets [9], [10], [11], [12], [13]. However, to date, there are no studies of anticipatory reactions to others’ *actions* directed to the self. The present study thus explores 2 to 4 month-old infants’ anticipatory adjustments to one infant-directed adult action - picking them up.

One measure of anticipatory responses to actions directed to self is postural adjustment. There is a sizeable literature on anticipatory postural adjustments prior to solitary actions, such as independent sitting [14], pulling self to standing [15], or reaching for an object [16], [17]. These studies have supported two parallel arguments: one, that motor behaviour necessarily involves prior planning and therefore the potential awareness of the impending action [18], and two, that postural adjustments are more effective for smooth action if completed prior to, rather than in response to, de-stabilising events [15], [19]. However, there is little research on anticipatory adjustments to *inter*-dependent action – that is, in response to the actions of another person that affect one’s postural stability. Studies using external perturbations to assess postural adjustments often use imbalance of supporting surfaces whilst supine or sitting [20], [21], [22] rather than the actions of other people. However, actions such as being picked up, being put on the shoulder to burp, being dressed and undressed or being examined in various ways, can and often do, de-stabilise posture much more drastically. Anticipatory rather than reactive adjustments to these actions must greatly aid infant postural comfort and smooth the interaction.

Being picked up is a common experience in infancy and anticipatory adjustments to it would be helpful for both infant and adult. Children with autism are reported by their parents to make no postural anticipatory adjustments to being picked up [23], suggesting that such anticipatory adjustments may be indicators of sensitivity to others’ intentions. This is supported by more general deficits in anticipation of others’ actions in the first year – such as in feeding situations –reported by studies using home movies of children later diagnosed with autism [24], [25]. When do typically developing infants anticipate and posturally adjust to being picked up? By 12 months infants lift up their arms as a request to be picked up and by 6 or 7 months of age infants may already be lifting their arms up in response to the approach of a parent [26], [27]. Anecdotal reports suggest that 4 and 5 month-olds arch their backs whilst being picked up, at least after the lift has begun. By 4 and 5 months of age infants are able to reach predictively [28] and clearly expect to be relieved from their distress when mothers approach, showing stillness while watching the approach even at 2 and 3 months [29]. However, the timing and specific nature of the emergence of postural adjustments to being picked up remains unclear. There are no systematic studies of the process in the early months nor any information about *anticipatory* (i.e., before the lift has begun, and perhaps even before physical contact) as distinguished from *responsive* postural adjustments (i.e., to the actual lift).

Research shows several developmental achievements in motor and attentional competence in the third month of life [30], [31], such as the onset of voluntary movements (including anti-gravity movements), the control of visual attention and binocular vision [32], [33] and a shift in infant postural control from body-oriented to space-oriented control [34], [35]. These findings suggest that a closer look at 2-, 3- and 4-month-old infants is crucial in terms of understanding anticipatory motor adjustments.

In the present set of studies we examined anticipatory responses to being picked up in two- to four-month-old infants in a relaxed chatting situation when they were not desperate for – but were open to – being picked up. In order to be picked up with ease and with minimal postural de-stabilisation, greater body rigidity (as e.g., in yogic postures and ballet) and decreased variability in posture [36] could be most useful. Thus, as indicators of preparation for being picked up, we looked for changes in thrashing or general rhythmic movements and in specific behavioural adjustments such as stiffening of the legs and arms through extension or flexion prior to being lifted up. We contrasted a period of prior Chat with two periods of potential anticipation before the lift – during the Approach of the mother’s arms before they contact the infant’s body, when any anticipation would be in response to the visual information, and after Contact but before they start to lift the infant, when any anticipation would be in response to the tactile information as well. Study 1 was an in-depth exploration of the anticipatory responses of eighteen 3-month-old infants, while Study 2 was a longitudinal exploration of developmental changes in these responses in ten infants at 2, 3 and 4 months of age.

## Methods

### Apparatus

We used a sensor mat (47 cm×47 cm) consisting of a 32×32 grid of 1056 pressure sensors (Tekscan) with a sampling rate of 20 Hz. The mat was placed on a plastic changing mat on a low table (36 cm off the floor) and recorded pressure from the infant’s body. Additionally, interactions were filmed with a digital camera focused on the infant (recording at 30 frames per second) and directly synchronized with the pressure mats.

### Procedure

The present study was approved by the Departmental Research Ethics Committee and written consent was obtained from mothers before commencing the study. This procedure was part of a larger study involving exploration of picking up in different situations. Prior to the start of the testing session each mother was asked whether their infant at this age appeared to be showing any anticipation of their actions generally and, more specifically, of impending pick up in various situations. Mothers were then instructed to place the infants on the mat, chat with them and pick them up a few times during the interaction when they felt the infants were comfortable and attentive, ensuring that the infants could see their arms as they approached to pick them up.

### Measures

#### Extracting pick-up episodes

Mothers attempted between two and five pick up episodes in each session. However, several episodes had false starts or prolonged hesitations, and thus were excluded. For the remaining episodes, three criteria were used by two independent judges to ensure their usability: (i) the mother’s arms were approaching frontally and were therefore potentially visible to the infant; (ii) the infant’s gaze was directed towards the mother; and (iii) the episode was preceded by a period of engagement, increasing the likelihood of the infant wanting to be picked up. In one third of the sessions at each age more than one episode met these criteria and the first good episode was chosen. There was disagreement about the criteria in 5 cases which was resolved following re-viewing of the episodes.

#### Identifying phases within the pick-up episodes

Three phases were identified for each pick-up episode: (1) *Chat*: beginning from 5 seconds before Approach; (2) *Approach*: beginning from the onset of the mother’s arms starting to approach the infant until Contact; (3) *Contact*: beginning from the onset of the mother’s hands contacting the infant’s chest until the onset of the mother lifting the weight of the infant. Two independent coders viewed all episodes and identified the frame points for the onset of Approach, onset of Contact and onset of Lift. Initial coefficients of agreement (within 10 video frames, i.e., at 30 fps, 1/3 of a sec) were.84 for onset of Approach,.76 for onset of Contact, and.92 for lift. All disagreements were viewed by a third coder and resolved through discussion until100% agreement was reached on a second round of judgements; in all cases the earliest identified frame (within the 10 frame agreement space) was taken as the agreed point of onset.

#### Behavioural coding

Infant behaviour in the three phases in all episodes was coded by two coders naïve to the rationale for the study. Episodes were watched at least twice: initially at normal speed to identify relevant behaviours, and then frame by frame to identify onset and offset points of behaviours. Two measures were extracted for all behaviours: (a) Presence/Absence of the behaviour in each phase and, (b) Duration of the behaviour in each phase (relative to the duration of each phase in each infant). Initial inter-coder reliability coefficients are presented below: Cohen’s *kappa* was calculated for Presence/Absence of behaviour and Coefficients of Agreement (within 10 frames, with the mid-point used for analyses) were calculated for durations of behaviours. All disagreements were discussed and 100% agreement reached. Onset and offset frames were identified for periods of *Thrashing/General Movements*, (i.e., definite rhythmic movement of arms and/or legs, often indicating excitement, or less vigorous but regular general movements of arms and/or legs) in each phase. Initial inter-coder reliability for Presence/Absence was.92 and for Durations was.78. Onset and offset frames for *Specific Adjustments* were identified as behavioural shifts from the normal position (i.e., continuing body positions were not coded as Specific Adjustments) in four body regions: *Head*: turning to the side or lifting; *Chin*: tensing or lifting up, the latter often accompanied by arching of the back; *Arms*: widening out, raising up, or lifting back beside the head; *Legs*: extending flat and raising slightly upwards, or tucking up. Initial inter-coder reliability for Presence/Absence was.74 and for durations was.81. Additionally, during the Approach phase we coded the duration of infant gaze, as being directed *to Mother’s Face*; *to Mother’s Hands;* and *Away* (anywhere other than mother’s face or hands). Initial inter-coder reliability for Presence/Absence was.96 and for Durations was.87.

#### Ratings of motor maturity

To assess infant motor maturity two independent coders rated neck lag on lift and overall muscle tone. Neck lag on lift was rated when the infant’s body was at a 20° angle (using *Dartfish* software to measure angle of lift) on a 5-point scale (ranging from neck dropping backwards to the neck held in line with or lifted above the shoulders) with an inter-rater reliability of.94. Overall muscle tone was judged on a 5-point scale as an overall score during the episode (ranging from floppy and hypotonic to strong tone) and achieved an inter-rater reliability of.85.

#### Pressure mat data

Infant weight on the mat (force rather than pressure) in three regions – head, upper and lower back – was analysed to identify whether the onset of Approach or onset of Contact led to any global shifts in postural organisation. The collected time series of force data were analysed using Recurrence Quantification Analysis (RQA) which quantifies aspects of the temporal evolution of a time series, such as its predictability, variability, or repetitiveness [37], [38]. For the present investigation, we examined the percentage of recurrent temporal structure in postural activity (%RECurrence) [38]. That is, any infant adjustment of posture would be revealed by changes or disruptions in force, quantified by %REC, with a drop in %REC indicating an increase in the structure of postural variability (i.e., showing a shift from one state to another) and an increase in %REC indicating a decrease in the structure of postural variability (i.e., showing stability of postural state). We looked for shifts in postural stability between three consecutive seconds in all three phases – Chat, Approach and Contact. We would expect systematic shifts in posture during the Approach and Contact phases, but not during the Chat phase. In the Chat phase we investigated changes in infants’ postural activity in the 1^st^, 2^nd^ and 3^rd^ seconds of Chat. In the Approach and Contact phases we investigated changes in infants’ postural activity in three intervals: (a) before (−1.5 to −0.5 s), during (−0.5 to 0.5 s), and after (0.5 to 1.5 s) the onset of the Approach phase, and (b) before (−1.5 to −0.5 s), during (−0.5 to 0.5 s), and after (0.5 to 1.5 s) the onset of the Contact phase. The length of the intervals (i.e., 1 s) was chosen so that enough data points for analysis were available, but also that the chosen intervals were still temporally close to the onset of Approach or the onset of Contact. The intervals were sufficient to reveal significant decreases between each consecutive second in distance of the mother’s hands (using the hand nearest the camera) to infant chest (measured using the *Dartfish* software) during the Approach phase but no differences in the Chat phase. ANOVAs at each age between the segments in the Approach phase revealed *p*-values ranging from *p*<.001 to <.03 and for the Chat phase from *p*<.46 to <.89.

## Study 1

### Participants

Data from eighteen 3-month-old infants (*M* = 3; 4.8 days, *SD* = 3; 4.9 days, range = 3;0 to 3;15; 5 girls) and their mothers were analysed for Study 1. All infants were healthy, full-term (at least 37 gestation weeks), from lower- to middle-class families, predominantly British (89%) and Caucasian (94%).

### Results and Discussion

#### Behavioural changes

**Figure 1:**
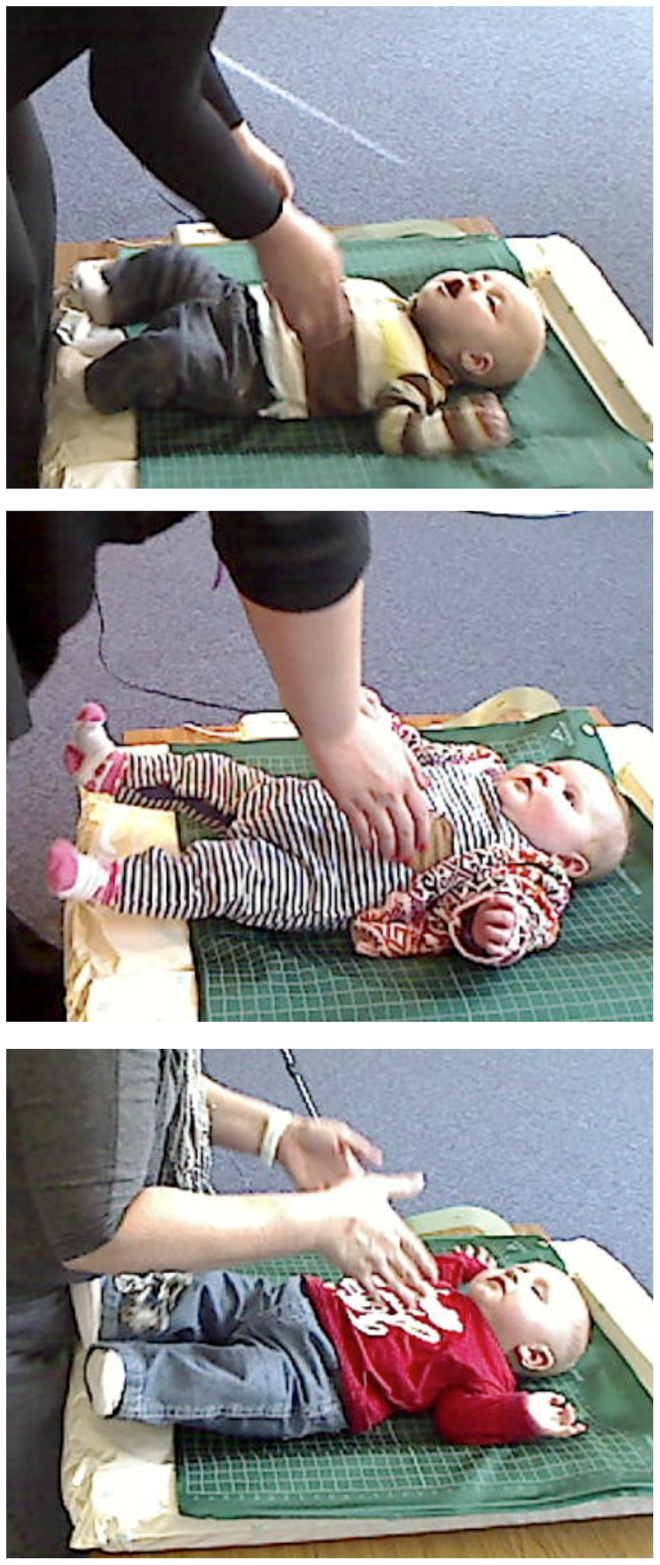
Illustrations of Common Arm and Leg Adjustments During Approach at 3 Months. Note: (a) Infant LU: Mother’s Arms approach above the infant’s chest. Infant Arms still and up and back; Chin raised (back arched); Legs still. (b) Infant VI: Mother’s Arms approach above the infant’s chest. Infant Legs still, extended and slightly raised; Arms widened outwards. (c) Infant TO: Mother’s Arms approach above the infat’s chest. Infant Arms still and up and back; Legs extended and slightly raised. Participants have given written informed consent, as outlined in the PLOS consent form, to publication of their photographs.

**Figure 2:**
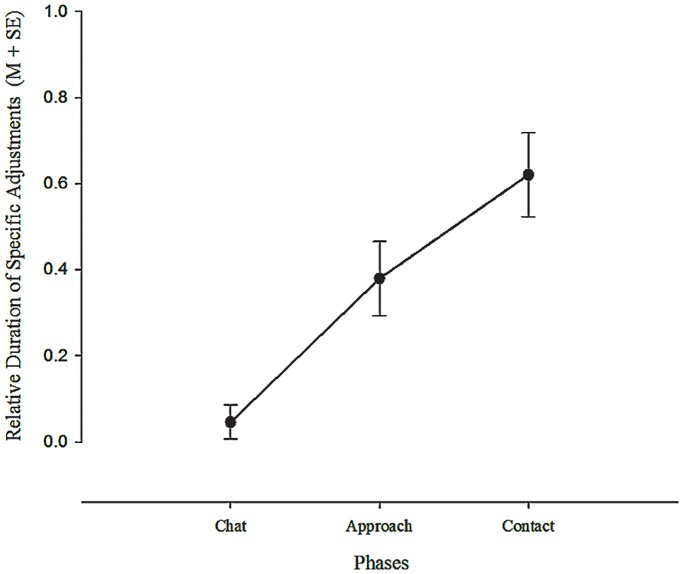
Durations of Specific Adjustments Across Phases at 3 Months. Note: Means in all Phases significantly different from each other to *p*<.014.

The most common Specific Adjustments made by infants were of the Arms (present in 14 infants) and the Legs (present in 12 infants); Head and Chin adjustments were less common (present in 6 and 3 infants respectively). Figure 1 illustrates some typical adjustments of the Arms and Legs (see also Movie S1). The Presence of adjustments differed significantly between the phases, χ^2^(2) = 17.2, *p*<.0001, with only 2 infants showing adjustments during Chat, 12 during Approach and 14 during Contact. There was a significant differentiation in the Presence of Specific Adjustments between the Chat and Approach phases, χ^2^(1) = 9.47, *p* = .0021 but not between Approach and Contact. Thus, infant adjustments were prompted principally by the onset of the mother’s arms approaching the infant, rather than the contact with the infant’s body. Only 3 of the 18 infants showed no Specific Adjustments in any phase. A repeated-measures ANOVA computed for comparing the Durations of Specific Adjustments in each Phase (Chat, Approach, Contact) revealed a significant main effect of Phase, *F*(2,34) = 18.01, *p*<.0001, η^2^ = .51, with a significant linear trend, *F*(1,17) = 34.40, *p*<.0001, η^2^ = .669, as can be seen in Figure 2. Pairwise comparisons revealed significant differences between Chat and Approach (*p = *.004) and between Approach and Contact (*p* = .014).

The Presence of Thrashing/General Movements also differed significantly between phases, χ^2^(2) = 12.4, *p* = .002, with a significant decrease between the Approach (13 infants) and Contact (3 infants) phases, χ^2^(1) = 9.11, *p* = .003, but not between Chat (11 infants) and Approach. Thirteen (of the 18) infants showed Thrashing/General Movements at some point: 9 infants during both Chat and Approach phases but not after Contact; 3 infants during all three phases and 1 infant only during Approach. The Presence of these movements during the Chat as well as the Approach phase may reflect infants’ excitement at an anticipated pick-up (or the hope for one) prompted by the mother’s preparatory body movements during the Chat phase even prior to the Approach of her arms. A repeated measures ANOVA on the relative Durations of Thrashing/General Movements as a function of Phase (Chat, Approach, Contact) also showed a significant effect of Phase (*F*(2, 34) = 10.644, *p* = .0003, η^2^ = .39) with a significant linear reduction in durations (*F*(1,17) = 15.83, *p* = .001, η^2^ = .48). Pairwise comparisons revealed a significant reduction between Approach and Contact (*p* = .011) and nearly significantly between Chat and Approach (*p* = .051).

#### Motor maturity

Chi-square tests revealed no association between infants who made Specific Adjustments and those with high versus low neck control or high versus low muscle tone (in both cases *p* = .64).

#### Global postural change

In order to look at global postural changes at the period when Specific Adjustments increased (i.e., during the Approach phase) and at the period where Thrashing/General Movements decreased (i.e., during the Contact phase), we analyzed the %RECurrence values for 3 seconds of the force profiles of the Chat, Approach and Contact phases, as described earlier. We also examined the possibility of different force profiles in three different body regions – head, upper back and lower back.

**Figure 3:**
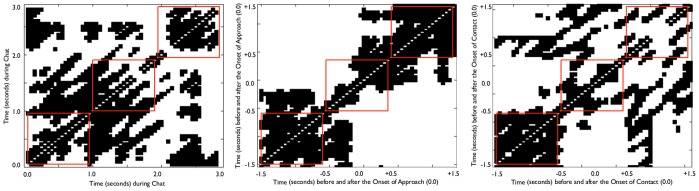
Illustrative Example of Recurrence Force Profiles for (a) Chat, (b) Approach and (c) Contact Phases. Note: The Recurrence Force Profiles show the sum of individual recurrence points (using the measure of %RECurrence). The more saturated parts of the plots show a higher density of recurrence points (i.e., the small black dots). Areas of higher density indicate greater sameness of posture (which could either be continuing stillness or continuing small scale movements). Areas of low saturation show little recurrence and could either indicate irregular movements or large scale movements. In Figures (b) and (c), 0.0 on the axes marks the approximate point of Onset of Approach or Onset of Contact (because each dot in the plot consists of several data points in the time series, the absolute point of Onset cannot be precisely located). The three red squares in each figure highlight the three 1 second time segments of analysis in each plot.

Preliminary analyses showed no main effect of body region or any interaction of this factor with other variables; thus, body region was dropped from all subsequent analyses. Consequently, three repeated measures ANOVAs were computed separately for the Chat phase, the Approach phase and the Contact phase, with Time (the 1^st^, 2^nd^ and 3^rd^ segments) as the within-subjects factor. For the Chat phase, there was no effect of Time, *F*(2, 102) = .82, *p* = .449, η^2^ = .015, showing no change in %RECurrence (see Figure 3a). For the Approach phase, there was a marginally non-significant effect of Time, *F*(2, 102) = 2.78, *p*<.071, η^2^ = .100, showing a drop in %RECurrence immediately after the onset of Approach, followed by an increase. As can be seen in Figure 3b, the high recurrence (strong clustering of dots along the diagonal) at the start of the profile dissipates into a pattern of high variability, then re-forms into a strong pattern within 500 ms after the onset. For the Contact phase, there was a significant main effect of Time, *F*(2, 102) = 16.73, *p*<.001, η^2^ = .246. As can be seen in Figure 3c there was a decrease in %REC in the third time segment (between.5 and 1.5 seconds after onset of Contact). These results showed that the onset of Approach leads to an immediate response, with the infant shifting to a different postural activity, while in response to the onset of Contact there is an initial stillness (in the first 500 ms after contact) before there is an increase in variability in the third time segment.

In summary, Specific Adjustments were present in 12 of 18 three-month-olds during Approach and in a further 3 infants only after Contact, with a reduction in Thrashing/General Movements in 13 infants, particularly after Contact. From the force data, shifts in postural stability were evident immediately after onset of Approach and more slowly after Contact, with no regular shifts during Chat. Thus, 3-month-olds show appropriate anticipatory adjustments to the Approach of the mother’s arms before they are picked up.

## Study 2

### Participants

Ten (3 girls) of the 3-month-old infants from Study 1 were additionally observed at 2 months (*M* = 2;6.6 days, *SD* = 2;50 days, range = 2;2 to 2;18) and at 4 months (*M* = 4;3.1 days, *SD* = 4;3.8 days, range = 3;28 to 4;8).

### Results and Discussion

#### Longitudinal behavioural changes

A Chi-Square test showed no age differences in the Presence of Specific Adjustments. At 2 months, however, although all ten infants showed Specific Adjustments, their Presence showed no significant differentiation between the Chat phase on the one hand and either the Approach or Contact phases on the other (*p* = .303) with 5 infants showing Adjustments in *all* three phases. However, such a differentiation was significant at 3 months, χ^2^(1) = 7.27, *p* = .007, and at 4 months, χ^2^(1) = 5.21, *p* = .023. At all ages, the most common adjustments were of the Legs and Arms. A repeated measures ANOVA on the relative Durations of Specific Adjustments with Age (2, 3, 4 months) and Phase (Chat, Approach, Contact) as within-subjects factors revealed a main effect of Phase, *F*(2,36) = 11.74, *p = *.001, η^2^ = .566 (see Table 1). Pairwise comparisons revealed significant increases between the Approach and Contact Phases (*p* = .006), between the Chat and Contact Phases (*p = *.003), and a nearly significant difference between the Chat and Approach Phases (*p* = .081). There was no main effect of Age or any interaction between Age and Phase.

**Table 1:** Descriptive Statistics (*M*, *SD*) for Proportional Durations of Specific Adjustments and Thrashing/General Movements at all Ages.

	2 months	3 months	4 months
	Specific Adjustments		
Chat	.272 (.268)	.011 (.036)	.240 (.395)
Approach	.435 (.440)	.330 (.377)	.243 (.284)
Contact	.634 (.420)	.546 (.447)	.596 (.471)
	Thrashing/General Movements		
Chat	.775 (.323)	.585 (.437)	.493 (.414)
Approach	.570 (.395)	.300 (.269)	.310 (.272)
Contact	.207 (.337)	.156 (.345)	.149 (.253)

Although with the small sample size the null difference between ages must be treated with caution, the presence of specific adjustments even at 2 months of age needs to be taken seriously. Given that 3-month-old infants, if they have received sufficient experience with an action, can distinguish the goals of actions directed to objects [5], the present finding of anticipatory responses even at 2 months to a familiar maternal action is plausible and consistent. However, the lack of association with Phase of the Presence of Specific Adjustments at 2 months suggests an incoherence in the responses, with the infants showing early anticipatory adjustments but not finely tuned in to the progress of the mother’s actions.

The Presence of Thrashing/General Movements was significantly associated with Phase at all ages: at 2 months, χ^2^(2) = 7.50, *p* = .024, 3 months, χ^2^(2) = 6.70, *p* = .035, and at 4 months, χ^2^(2) = 5.83, *p* = .054, with lower Presence in the Contact phase at all ages. A repeated measures ANOVA on the relative Duration of Thrashing/General Movements with Phase (Chat, Approach, Contact) and Age (2, 3, 4 months) as within-subjects factors showed a main effect of Phase, *F*(2,36) = 20.551, *p* = .000023, η^2^ = .695, with pair-wise comparisons revealing significant decreases in Duration between Chat and Approach (*p* = .002), between Approach and Contact (*p* = .019), and between Chat and Contact (*p* = .0003; see Table 1). There was no main effect of Age or any interaction between Phase and Age.

#### Infant gaze during approach

**Figure 4:**
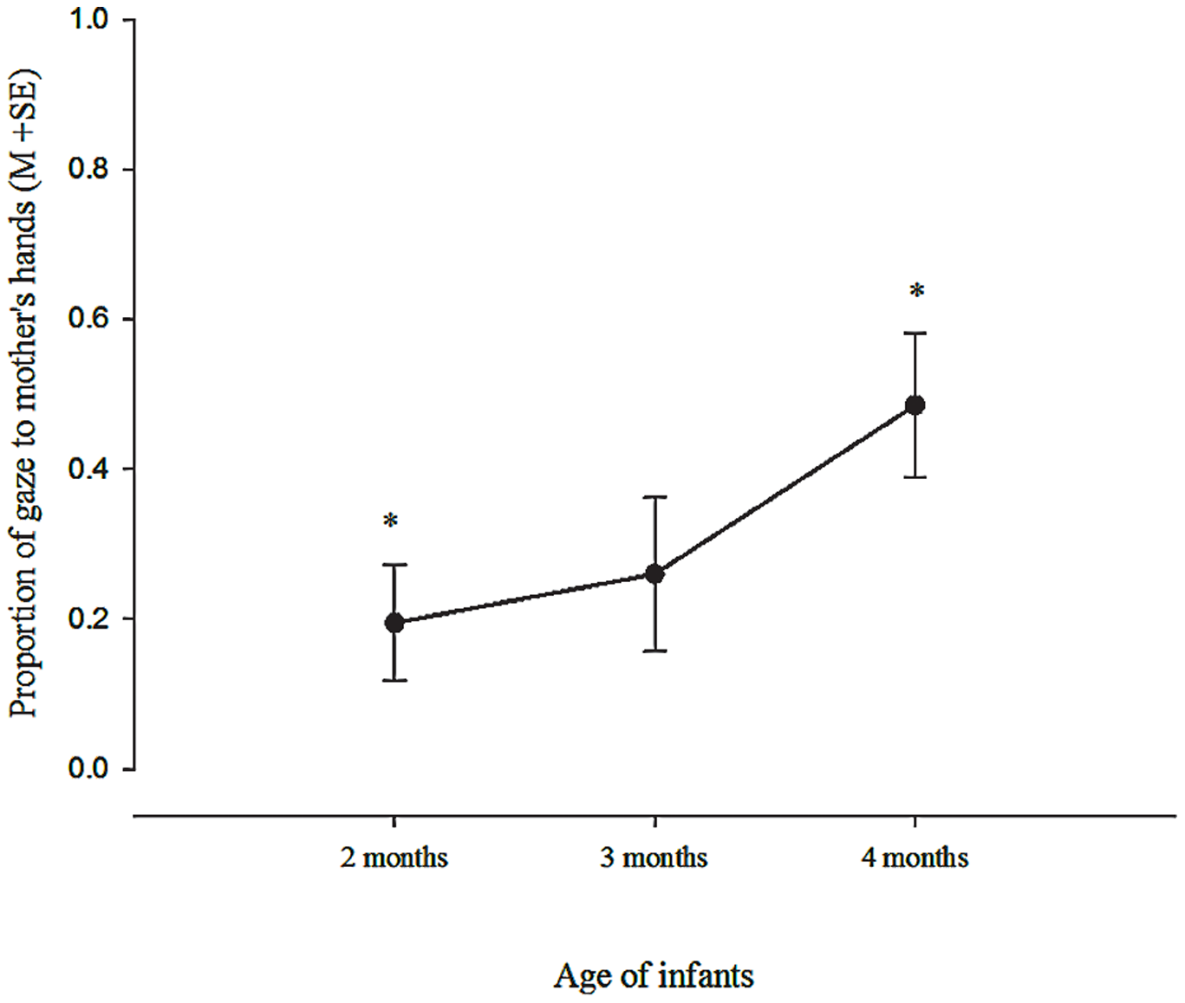
Relative Duration of Gaze to Mother’s Hands During Approach. Note: Means with asterisks significantly different from each other to *p*<.01.

The largest proportion of gaze during Approach was directed to Mother’s face (*M = *.69, *SD* = .30, range:.21–1.00), with very little gaze Away (*M* = .03, *SD* = .10, range:.0–.49) with no significant age differences in either. There was a significant linear trend, however, in proportion of gaze to Mother’s Hands, *F*(1,9) = 8.11, *p* = .019, η^2^ = .474, with a significant increase between 2 and 4 months (*p* = .019, see Figure 4; at 2 months *M* = .17, *SD* = .22, range:.0 -.58; *M* = .26, *SD* = .30, range:.0 -.74; *M* = .45, *SD* = .29, range:.0 -.79). The increase in interest in the mother’s hands may follow the well-known phenomenon of watching their own hands after 2 months [39] and reveal a more general interest in hands *per se* [40]. This interest in the hands, however, may constitute a distraction from, rather than a help in, adjusting to being picked up.

#### Global postural changes over age

As in Study 1, preliminary analyses showed no main effect of body region or any interaction of this factor with other variables; thus, body region was dropped from all subsequent analyses. Consequently, three repeated measures ANOVAs were computed separately for the Chat phase, the Approach phase and the Contact phase, with Time (the 1^st^, 2^nd^, and 3^rd^ segments) and Age (2, 3, 4 months) as the within-subjects factors. Two participants were not included in this analysis, because the Approach phase was too rapid to provide enough data points.

For the Chat phase there was a significant main effect of Age, *F*(2, 54) = 5.27, *p* = .008, η^2^ = .154, showing a general increase in %REC with age of the infants. Pairwise comparisons revealed a significant increase in %REC from 2 to 4 months, *F*(1, 29) = 9.07, *p* = .005, η^2^ = .238, but not between 2 and 3 months (*p* = .111) or between 3 and 4 months (*p* = .093). For the Approach phase there was a significant main effect of Time, *F*(2, 46) = 6.72, *p* = .003, η^2^ = .226, showing an immediate drop in %REC after the onset of Approach, followed by an increase - a similar pattern to that in Study 1. However, there was a significant interaction between Time and Age, *F*(4, 46) = 2.71, *p* = .035, η^2^ = .105, with pairwise comparisons showing a significant decrease in force at Approach only for 4-month-old infants, *F*(2, 52) = 8.803, *p* = .001, η^2^ = .253. For the Contact phase, there was also a significant main effect of Time, *F*(2, 52) = 22.99, *p*<.001, η^2^ = .469, with infant postural activity remaining stable immediately upon Contact with variability increasing later, again as in Study 1. There was also a significant interaction between Time and Age, *F*(4, 52) = 2.67, *p* = .036, η^2^ = .093. Pairwise comparisons revealed a significant decrease in force in the 3^rd^ time segment for 3-month-olds, *F*(2, 58) = 11.925, *p*<.001, η^2^ = .291, and 4-month-olds, *F*(2, 58) = 14.520, *p*<.001, η^2^ = .334 but not for 2-month-olds. Thus patterns of change over time in %RECurrence were not significant for 2-month-olds either for the onset of Approach or for the onset of Contact.

#### Maternal reports of anticipation

At 2 months, 5 of the 10 mothers reported some signs of anticipation of being picked up – but their reports were hesitant and the behavioural changes reported were limited to excitement or quieting, with only one mother reporting anticipatory arm raising. By 3 months, mothers reported much clearer signs of anticipation, with 6 of the 10 mothers reporting specific behavioural adjustments, 4 of these referring to tensing of back or body. By 4 months, 9 of the 10 mothers reported specific anticipatory adjustments, with 4 reporting tensing. Although we cannot tell from these reports how much the mothers’ reports were a function of taking part in the study and the resulting focused attention, in almost all cases the maternal reports referred to fewer and less subtle behavioural adjustments than the video analyses revealed.

In sum, Study 2 showed that Specific Adjustments were present at all ages, but were less differentiated between phases at 2 months. At all ages Thrashing/General Movements reduced during the pick up, particularly after Contact. Global postural shifts were also more phase differentiated in the older infants with no significant effects of time in the 2 month-olds either for Chat or for the onset of Approach or Contact.

## General Discussion

This paper is the first to examine anticipatory postural adjustments by infants to the potentially de-stabilising actions of other people on the infant’s body. Thus far, studies of anticipatory postural adjustments have been restricted to the infant’s anticipation of their own actions, reported from around 6 or 10 months of age [14], [15], and infant anticipatory gaze to others’ actions on objects has been reported from 6 months of age [6]. The data reveal two principal findings with serious theoretical and methodological implications: one, that from as early as 2 months of age infants show specific postural adjustments to being picked up, even before there is physical contact; and two, that developmental changes in anticipatory postural adjustments between 2 and 4 months appear to be a matter of increasing smoothness and coordination within the phases of the pick-up rather than of the development of new types of response.

Study 1 showed that the majority of 3 month-olds reduced Thrashing/General movements and made Specific Adjustments during the Approach of the mother’s arms. These behaviours can serve to help the smoothness of the pick-up in three ways: increasing body rigidity, creating space for the mother to hold the infant’s chest and reducing the likelihood of the head flopping back after the lift. Rigidity of the body can be enhanced by greater stillness (the reduction in Thrashing/General Movements especially after Contact) and by greater stiffening of the extremities (the occurrence of Leg Extending/raising or Leg Tucking up and Chin Raising). The Arm widening/raising/lifting back (prior to contact) all served to create a more comfortable space for the mother to grasp the infant’s chest. And lastly, the rotation in Head Turning may have served to increase torque in the neck muscles thus reducing the lag of the head. The rapidity of the occurrence of these behavioural adjustments in response to Approach was supported by evidence of the global postural shifts, with the infant’s posture changing rapidly at the onset of Approach, and then steadying before changing again more slowly after Contact.

Study 2 showed that even at 2 months infants showed the same types of Specific Adjustments as at 3 and 4 months, with similar patterns of decreasing duration of Thrashing/General movements and increasing duration of Specific Adjustments in each phase. However, the Presence of Specific Adjustments was significantly differentiated by Phase (less during Chat and more during Approach and Contact) only in the 3- and 4-month-olds and not in the 2-month-olds. Similarly, the recurrence analyses showed that only at the older ages was the size of the global postural shift in force significantly related to time either at onset of Approach or at onset of Contact. In sum, 2-month-olds showed similar anticipatory adjustments to the 3- and 4-month-olds, but were less clearly attuned to the mother’s actions in terms of timing, often showing adjustments too early. This developmental increase in temporal coordination could be explained by one or both of two factors: a) a clearer grasp of the temporal course of the approaching arms in the 3 and 4 month-old infants, and b) better motor coordination at 3 and 4 months than at 2 months, supporting previous theorising [34], [35], [41]. Whatever the explanation, the view that only from around 6 months do infants enter the phase of ‘secondary variability’ allowing them to adapt their behaviour in more than a minimal way to external situations seems problematic in the light of the present findings [31]. The postural adjustment to specific situations and external conditions seems fairly complex long before 6 months of age.

These findings have two theoretical implications. First, they suggest that others’ intentional actions directed to the self may be simpler to grasp and anticipate than actions directed to other objects. Second, and most importantly, they show that active participation in joint action evidenced by infant adjustments to maternal actions is present very early in life suggesting that such participation may well be foundational for further development of intention awareness.

The goal-directedness in others’ actions directed towards oneself, i.e., in second-person interactions, generally require some sort of response and thus have a different phenomenal quality to actions directed towards other things or other people [13], [42], [43], [44]. Thus, infant grasp of the goals of actions directed towards themselves may be easier than of actions directed elsewhere. While anticipation of the goals of externally directed actions has been found at 6 months of age [6], the current findings suggest that the anticipation of the goals of infant-directed actions is present by 2 months of age and very clearly by 3 months of age with action-appropriate bodily adjustments. This interpretation is supported by findings in infant attention-awareness, showing appropriate emotional reactions to attention to self before reactions to attention directed to external targets [12]. If habituation and looking-time measures (with less stringent demands than anticipation measures) [1] were to be used for measuring the awareness of infant-directed actions, from the present findings one would predict that this awareness would be found even earlier, possibly not long after birth. This prediction is supported by evidence from looking preference studies in the first month or even shortly after birth [9], showing infant awareness of gaze to self.

An alternative interpretation to that of the awareness of goal-directedness could be that the infants are merely associating an impersonal (non-psychological) event with its outcome, thus adjusting to the approaching arms to enhance comfort. Associations between initial signals and outcomes are clearly necessary for any anticipation (for infants or for adults). However, the predominant gaze to the mother’s face suggests at least that the stimulus event was not seen by the infant as an ‘impersonal’ event, but one associated with the mother’s agency. It may be more helpful to conceive of intentions and goal-directedness as embodied, and therefore perceivable, in actions (that is, as characterising and differentiating them) [13], [43], [44], [45] than as mental states hidden behind actions (and therefore needing inference for grasping them) [46], [47]. Such a theoretical shift would allow us to describe the early ways in which the perception of intentions and goals allows (and perhaps requires) participation in joint actions before infants have the conceptual ability to represent them, and the ways in which any problems in these early participations [23], [48] may further affect the developing understanding of intention. To further understand the nature of this infant participation we still need to know the extent to which infants (a) discriminate different kinds of actions directed to the self, (b) respond to unfamiliar actions directed to the self, and (c) are influenced in their discrimination and responses by different maternal styles of acting towards infants.

These findings suggest a methodological re-think on three issues. First, if actions directed to the self do hold a privileged position in revealing infant awareness of the goal-directedness of actions, then current research in infant social cognition needs to actively use participatory rather than spectatorial methods of investigation. Second, if familiar and real-life actions and situations reveal greater infant awareness than novel actions and situations, then using the familiarity rather than avoiding it as a contaminating factor may teach us more about the early stages of awareness. Third, infant awareness of intentionality may itself be seen as embodied, and thus available to analysis in the form of motor adjustments in joint action, allowing a richer form of experimentation [49], [50], [51].

In sum, the present findings show that the real-time anticipation of others’ actions upon the self is an early achievement in infancy, and that even by 2 months of age these anticipations are acted upon by appropriate bodily adjustments which, by 3 and 4 months of age assist in the smooth coordination of the impending action. The infant is thus actively participating in joint actions from very early in life. Unless one adopts a preformationist model of awareness, infant participation in such joint actions must constitute and contribute towards the developing awareness of the intentional meaning of others’ actions, with the absence of such participation posing a marker of specific developmental dysfunction.

## Supporting Information

Movie S1 **Exemplar Pick up Episodes at 3 Months from which Stills in** **Figure 1** **were taken.** Note: (a) Infant LU. (b) Infant VI. (c) Infant TO.
(WMV)

## References

[1] Cannon E, Woodward A (2012) Infants generate goal-based action predictions. Dev Sci 15(2): 292–298. 22356184 10.1111/j.1467-7687.2011.01127.x PMC3612028

[2] Gredeback G, Melinder A (2010) Infants’ understanding of everyday social interactions: A dual process account. Cognition 114(2): 197–206. 19800056 10.1016/j.cognition.2009.09.004

[3] Woodward AL (1998) Infants selectively encode the goal object of an actor’s reach. Cognition 69: 1–34. 9871370 10.1016/s0010-0277(98)00058-4

[4] Woodward AL (1999) Infants’ ability to distinguish between purposeful and non-purposeful behaviors. Inf BehavDev 22: 145–160.

[5] Sommerville J, Needham A, Woodward A (2005) Action experience alters 3-month-old infants‘ perceptions of others‘ actions. Cognition 96(1): B1–B11. 15833301 10.1016/j.cognition.2004.07.004 PMC3908452

[6] Falck-Ytter T, Gredeback G, von Hofsten C (2006) Infants predict other people's action goals. Nat Neurosci 9(7): 878. 16783366 10.1038/nn1729

[7] Kanakogi Y, Itakura S (2011) Developmental correspondence between action prediction and motor ability in early infancy. Nat Commun 2: 341. 21654641 10.1038/ncomms1342

[8] Rosander K, von Hofsten C (2011) Predictive gaze shifts elicited during observed and performed actions in 10-month-old infants and adults. Neuropsychologia 49(10): 2911–2917. 21722655 10.1016/j.neuropsychologia.2011.06.018

[9] Farroni T, Csibra G, Simion F, Johnson M (2002) Eye contact detection in humans from birth. Proc Natl Acad Sci USA 99(14): 9602–9605. 12082186 10.1073/pnas.152159999 PMC123187

[10] Farroni T, Massaccesi S, Menon E, Johnson M (2007) Direct gaze modulates face recognition in young infants. Cognition 102(3): 396–404. 16540101 10.1016/j.cognition.2006.01.007

[11] Reddy V (2000) Coyness in early infancy. Dev Sci 3(2): 186–192.

[12] Reddy V (2003) On being the object of attention: implications for self-other consciousness. Trends Cogn Sci 7(9): 397–402. 12963470 10.1016/s1364-6613(03)00191-8

[13] Reddy V (2008) How infants know minds. Cambridge: Harvard University Press. 273 p.

[14] Rochat P (1995) Self-sitting and reaching in 5- to 6-month-old infants. Inf Behav Dev 18(1): 53–68.

[15] Witherington D, von Hofsten K, Rosander K, Robinette A, Wollacott MH, et al (2002) The development of anticipatory postural adjustments in infancy. Infancy 3(4): 495–517.

[16] Von Hofsten C (1982) Eye–hand coordination in newborns. Dev Psychol 18: 450–461.

[17] Von Hofsten C (1984) Developmental changes in the organisation of prereaching movements. Dev Psychol 20(3): 378–388.

[18] Bernstein N (1967/1984) The coordination and regulation of movements. Oxford: Pergamon. 196 p.

[19] Von Hofsten C (1993) Prospective control: a basic aspect of action development. HumDev 36: 253–270.

[20] Hadders-Algra M, Brogren E, Forssberg H (1998) Development of postural control-differences between ventral and dorsal muscles? Neurosci Biobehav Rev 22(4): 501–506. 9595561 10.1016/s0149-7634(97)00036-5

[21] Hedberg A, Forssberg H, Hadders-Algra M (2004) Postural adjustments due to external perturbations during sitting in 1-month-old infants: evidence for the innate origin of direction specificity. Exp Brain Res 157(1): 10–17. 15024537 10.1007/s00221-003-1811-z

[22] Nashner LM (1976) Adapting reflexes controlling the human posture. Exp brain Res 26: 59–7. 964327 10.1007/BF00235249

[23] Kanner L (1943) Autistic disturbances of affective contact. Nerv Child 2: 217–250. 4880460

[24] Saint-Georges C, mAhdhaoui A, Chetouani M, Cassel R, Laznik M-C, et al (2011) Do parents recognise autistic deviant behavior long before diagnosis? Taking into account interaction using computational methods. PloS One 6(7): e22393. 21818320 10.1371/journal.pone.0022393 PMC3144901

[25] Brisson J, Warreyn P, Serres J, Foussier S, Adrien J-L (2012) Motor anticipation failure in infants with autism: a retrospective analysis of feeding situations. Autism, DOI: 10.1177/1362361311423385 10.1177/1362361311423385 22250193

[26] Lock A (1984) The emergence of language: On being picked up. In: Lock A, Fisher E, editors. Language Development. Beckenham: Croom Helm Ltd. 39–48.

[27] Service V (1984) Maternal styles and communicative development. In: Lock A, editor. Language Development. Kent: Croom Helm Ltd. 132–140.

[28] Bertenthal B, von Hofsten C (1998) Eye, head and neck control: the foundation for manual development. Neurosci Biobehav Rev 22(4): 515–520. 9595563 10.1016/s0149-7634(97)00038-9

[29] Lamb M, Malkin C (1986) The development of social expectations in distress-relief sequences: A longitudinal study. Int J Behav Dev 9: 235–249.

[30] Prechtl H (1984) Continuity and change in early neural development. In: Prechtl H, editor. Continuity of Neural functions from pre-natal to post-natal life. Clinics in Developmental Medicine, 94. Oxford: Blackwell. 1–15.

[31] Hadders-Algra M (2005) Development of postural control during the first 18 months of life. Neural Plast 12(2–3): 99–108. 16097478 10.1155/NP.2005.99 PMC2565464

[32] Atkinson J (1984) Human visual development over the first 6 months of life. A review and a hypothesis. Hum Neurobiol 3(2): 61–74. 6378843

[33] Braddick O, Atkinson J (1983) Some recent findings on the development of human binocularity: A review. Behav Brain Res 10(1): 141–150. 6357224 10.1016/0166-4328(83)90160-2

[34] Einspieler C, Marschik P, Prechtl H (2008) Human motor behaviour: origin and early postnatal development. J Psychol 216(3): 148–154.

[35] Prechtl HFR (1989) Development of postural control in infancy. In: von Euler C, Forssberg H, Lagercrantz H, editors. Neurobiology of early infant behavior. Wenner-Gren International Symposia Series, 55. London: The MacMillan Press. 59–68.

[36] Dusing S, Harbourne R (2010) Variability in postural control during infancy: Implications for development, assessment, and intervention. Phys Ther 90: 1838–1849. 20966208 10.2522/ptj.2010033 PMC2996511

[37] Marwan N (2011) How to avoid potential pitfalls in recurrence plot based data analysis? Int J Bifurcat Chaos 21: 1003–1017.

[38] Webber CL, Zbilut JP (2005) Recurrence quantification analysis of nonlinear dynamical systems. In: Accessed 2009 August Riley MA, Van Orden GC, editors. Tutorials in contemporary nonlinear methods for the behavioral sciences. Available: http://www.nsf.gov/sbe/bcs/pac/nmbs/nmbs.jep. 23: 26–96.

[39] White BL, Castle P, Held R (1964) Observations on the development of visually-guided reaching. Child Dev 35(2): 349–364. 14163542 10.1111/j.1467-8624.1964.tb05944.x

[40] Amano S, Kezuka E, Yamamoto A (2004) Infant shifting attention from an adult’s face to an adult’s hand: A precursor of joint attention. Infant Behav Dev 27(1): 64–80.

[41] Trevarthen C (1986) The development of intersubjective motor control in infants. In: Wade MG, Whiting HTA, editors. Motor Development in children: Aspects of coordination and control. NATO ASI Series. Dordrecht: Martinus Nijhoff. 209–261.

[42] Zahavi D (2006) Subjectivity and selfhood: Investigating the first person perspective. Cambridge: MIT Press. 280 p.

[43] Schilbach L, Timmermanns B, Reddy V, Costall C, Bente G, et al. (in press) Towards a second person neuroscience. Behav Brain Sci. 10.1017/S0140525X12000660 23883742

[44] Gallagher S (2001) The practice of mind: Theory, simulation or primary interaction? Journal of Consciousness Studies 8(5–7): 83–108.

[45] Leudar I, Costall A (2004) On the persistence of the ‘problem of other minds’ in Psychology: Chomsky, Grice and Theory of Mind. Theory Psychol 14(5): 601–621.

[46] Tomasello M, Carpenter M, Behne T, Moll H (2005) Understanding and sharing intetnions: The origins of cultural cognition. Behav Brain Sci 28: 675–735. 16262930 10.1017/S0140525X05000129

[47] Penn D, Povinelli D (2007) On the lack of evidence that non-human animals possess anything remotely resembling a ‘theory of mind’. Philos Trans R Soc Lond B Biol Sci 362: 731–744. 17264056 10.1098/rstb.2006.2023 PMC2346530

[48] Elsabbagh M, Volein A, Csibra G, Holmboe K, Garwood H, et al (2009) Neural correlates of eye gaze processing in the infant broader autism phenotype. Biol Psychiatry 65(1): 31–38. 19064038 10.1016/j.biopsych.2008.09.034

[49] Becchio C, Sartori L, Castiello U (2010) Toward You: The social side of actions. Psychol Sci 19(3): 183–188.

[50] Gallese V (2001) The roots of empathy: the shared manifold hypothesis and the neural basis of intersubjectivity. Psychopathology 36: 171–180. 10.1159/000072786 14504450

[51] Robertson S, Johnson S (2009) Embodied infant attention. Dev Sci 12(2): 297–304. 19143802 10.1111/j.1467-7687.2008.00766.x

